# Increased SARS-CoV-2 seroprevalence and spread of infection without awareness among healthcare workers through 2020–2022 in a Japanese medical center

**DOI:** 10.1038/s41598-023-32193-4

**Published:** 2023-03-27

**Authors:** Rie Kanamori, Yan Yan, Kanami Ito, Hiroshi Fukuda, Satoshi Hori, Takamasa Yamamoto, Gene Igawa, Kaori Saito, Yuki Horiuchi, Shuko Nojiri, Yuji Nishizaki, Yoko Tabe, Kazuhisa Takahashi, Toshio Naito

**Affiliations:** 1 Department of General Medicine, Juntendo University Faculty of Medicine, Hongo 2-1-2, Bunkyo-Ku, Tokyo, 113-8421 Japan; 2 Department of Safety and Health Promotion, Juntendo University, Tokyo, Japan; 3 Infection Control Science, Juntendo University Graduate School of Medicine, Tokyo, Japan; 4 Department of Clinical Laboratory, Juntendo University Hospital, Tokyo, Japan; 5 Department of Clinical Laboratory Medicine, Juntendo University Faculty of Medicine, Tokyo, Japan; 6 Medical Technology Innovation Center, Juntendo University Graduate School of Medicine, Tokyo, Japan; 7 Division of Medical Education, Juntendo University Faculty of Medicine, Tokyo, Japan; 8 Department of Research Support Utilizing Bioresource Bank, Juntendo University Graduate School of Medicine, Tokyo, Japan; 9 Department of Respiratory Medicine, Juntendo University Faculty of Medicine, Tokyo, Japan

**Keywords:** Epidemiology, Infectious diseases, Viral infection, Public health

## Abstract

Despite Japan’s high vaccination coverage, daily numbers of new COVID-19 cases have been high. However, studies on the seroprevalence among Japanese people and the causative factors for rapid spread have remained limited. In this study, we aimed to examine the seroprevalence and associated factors in healthcare workers (HCWs) of a medical center in Tokyo using blood samples drawn at annual check-ups from 2020 to 2022. We found that of the 3,788 HCWs in 2022 (by mid-June), 669 were seropositive for N-specific antibodies (tested by Roche Elecsys Anti-SARS-CoV-2 assay); the seroprevalence surged from 0.3% in 2020 and 1.6% in 2021 to 17.7% in 2022. Notably, our study found 325 (48.6%; 325/669) cases were infected without awareness. Among those with a previously PCR-confirmed SARS-CoV-2 infection during the past three years, 79.0% (282/357) were found after January 2022, after the Omicron variant was first detected in Tokyo at the end of 2021. This study indicates the fast spread of the SARS-CoV-2 among HCWs during the Omicron surge in Japan. The high percentage of infection without awareness may be a key driving factor causing rapid person-to-person transmission, as shown in this medical center with high vaccination coverage and strict infection control measures.

## Introduction

Coronavirus disease (COVID-19) caused by SARS-CoV-2 infection has spread continuously worldwide despite comprehensive vaccinations. The study reported by the Centers for Disease Control and Prevention (CDC) showed that three doses of mRNA vaccine, including BNT162b2 and mRNA-1273, had higher efficacy against SARS-CoV-2 infection than two doses or no vaccination during the Delta-predominant period and the period of Omicron emergence^1,2^. In Japan, more than 80% of Japanese people completed two doses of vaccine (mainly using mRNA vaccines) against SARS-CoV-2 (by the end of August, 2022)^3^. In addition, more than 60% of Japanese people had received three doses by the beginning of September 2022^3,4^. In particular, most Japanese people had continued to comply with public health measures, such as wearing masks and keeping social distancing, even after receiving booster doses. Despite vaccine administration efforts and compliance with public infection control measures, the number of COVID-19 cases surged rapidly after the Omicron variant emerged.

The Omicron variant is considered more transmissible than the Delta variant^5^. In addition, previous studies from November 2021 to April 2022 reported that the percentage of asymptomatic infections ranged from 23.0 to 46.7%; the percentage was higher in countries with high vaccination coverage compared to those with low coverage^6–9^. Moreover, a recent cohort serological study in the U.S., with more than 80% of participants having been given three doses of vaccine, reported that more than half of the Omicron infections were asymptomatic and/or occurred without awareness^10^. These previous studies showed that not only high transmissibility, but also infection without awareness, might cause further and wider transmission.

As the Omicron variant and its subvariants spread rapidly across Japan since early 2022, Tokyo has experienced a high number of daily COVID-19 cases^11^. Yet, studies of the updated prevalence and driving factors have remained limited. We previously reported the seroprevalence of nucleocapsid (N)-specific antibodies in HCWs in 2020 and 2021 at the time of annual health check-ups in Juntendo University Hospital (JUH) in Tokyo, Japan^12,13^. The seroprevalence in 2021 after the two rounds of mass vaccination, even after the Delta variant started to spread rapidly in the Tokyo metropolitan area, remained as low as 1.6%, which did not constitute a notable increase from 2020 (0.3%)^12,13^.

Because understanding of the seroprevalence and related factors is critical for public health policy-making, the aim of this study is to examine the seroprevalence of SARS-CoV-2 and infection without awareness among HCWs in our university hospital, a setting with a high vaccination rate and strict infection control measures. We analyzed serological samples from 2022 annual health check-ups (July 17 through August 21, 2022) and participating HCW records on vaccination status and previous PCR-confirmed COVID-19 infections.

## Results

A total of 3788 HCWs participated in this study. The basic characteristics of the participants (at the time of the 2022 annual check-up) are shown in Table 1. The median age was 36 years old [range 20–86]. More than 90% of the participants were younger than 60 years of age, and more than half of the nurses were 20–29 years of age (Supplementary Table S1). 62.8% of the participants were females. The majority of participants (89.3%) received three doses of COVID-19 vaccine, whereas only a few participants (0.3%) received four doses, in accordance with the criteria of four doses of vaccination in Japan (for those with medical conditions and senior citizens aged 65 years or older). COVID-19-dedicated staff included medical doctors, nurses, laboratory personnel, and paramedics (Supplementary Table S2).

**Table 1:** Basic characteristics and N-specific antibody results (positive vs. negative).

	Overall (%)^a^	N-specific antibody positive n (%)	N-specific antibody negative n (%)	*P* value
All	3788 (100.0)	669 (100.0)	3119 (100.0)	
Age (years)				
20–29	1092 (28.8)	237 (35.4)	855 (27.4)	< 0.001
30–39	1171 (31.0)	223 (33.3)	948 (30.4)	
40–49	829 (21.9)	147 (22.0)	682 (21.9)	
50–59	436 (11.5)	49 (7.3)	387 (12.4)	
60 or older	260 (6.9)	13 (1.9)	247 (7.9)	
Sex				
Male	1409 (37.2)	248 (37.1)	1161 (37.2)	0.965
Female	2379 (62.8)	421 (62.9)	1958 (62.8)	
Profession				
Medical doctors	1497 (39.5)	279 (41.7)	1218 (39.1)	< 0.001
Nurses	1080 (28.5)	238 (35.6)	842 (27.0)	
Laboratory personnel	182 (4.8)	18 (2.7)	164 (5.3)	
Paramedics	320 (8.4)	33 (4.9)	287 (9.2)	
Administrative staff	542 (14.3)	79 (11.8)	463 (14.8)	
Researchers	157 (4.1)	22 (3.3)	135 (4.3)	
Others	10 (0.3)	0 (0.0)	10 (0.3)	
No. of vaccine doses received				
Unvaccinated	80 (2.1)	18 (2.7)	62 (2.0)	< 0.001
1 dose	14 (0.4)	4 (0.6)	10 (0.3)	
2 doses	300 (7.9)	77 (11.5)	223 (7.1)	
3 doses	3384 (89.3)	570 (85.2)	2814 (90.2)	
4 doses	10 (0.3)	0 (0.0)	10 (0.3)	

^a^Numbers of participants by categories in this table indicate the status at the time of the 2022 health check-up, conducted from June 8 to June 20 in Juntendo University Hospital of Tokyo, Japan. A two-tailed *p* < 0.05 was considered significant.

**Figure 1:**
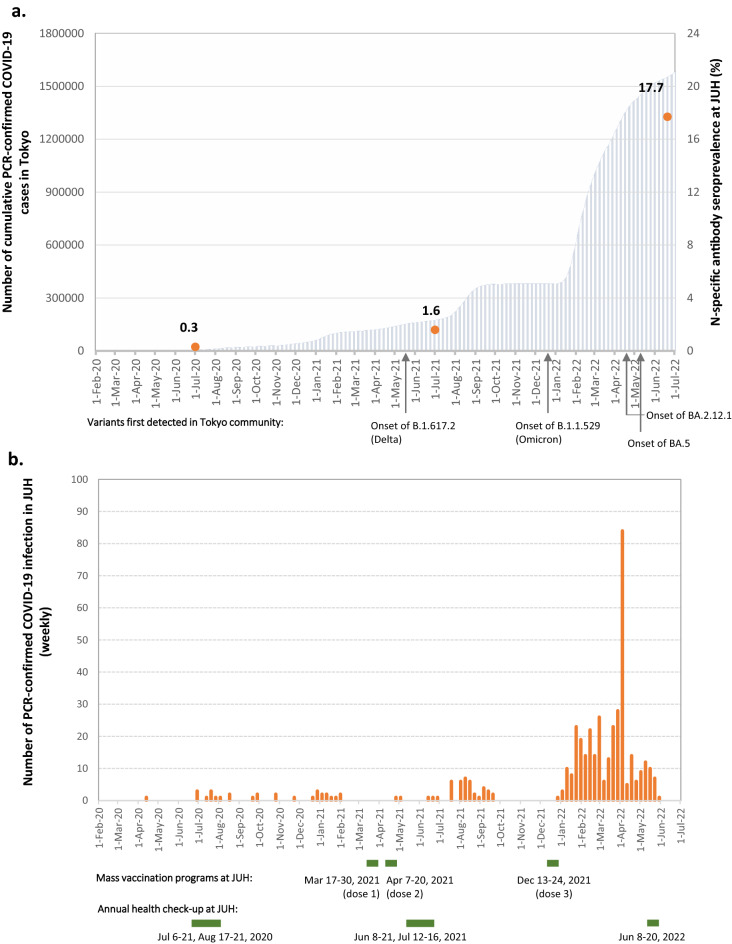
Infection rate at JUH with respect to the cumulative confirmed COVID-19 cases in the Tokyo metropolitan area. The number of PCR-confirmed COVID-19 infection cases until May 30, 2022 at JUH, including two participants with two positive PCR tests respectively—one on July 19 and September 11, 2020 and another on July 22, 2021 and January 28, 2022. Infection rates at JUH were calculated by the number of HCWs who were N-specific antibody-positive divided by the number of HCWs who participated in the annual health check-ups and gave consent for serological analysis. B.1.617.2 (Delta)^14^, B.1.1.529 (Omicron)^15^, BA.2.12.13^16^, and BA.53^16^ were detected via implementing variant screenings in Tokyo community. Mass vaccination programs were conducted for all JUH’s HCWs who were willing to receive COVID-19 vaccines. Although the majority of HCWs received vaccines during the campaign periods, some might have individually received them later.

The seroprevalence at the 2022 annual health check-up was 17.7% (95% CI [1.65–18.9]; 669/3788) (Table 1, Fig. 1a), increased from the previously reported 0.3% at year 2020 and 1.6% at year 2021^12,13^. There were significant differences in seroprevalence across age categories, profession categories, and vaccination status, but there was no significant difference between male and female participants (Table 1). The odds ratios (ORs) with respect to the basic characteristics: age, sex, profession categories, and vaccination status, and the OR of COVID-19 dedicated staff in the multivariable logistic regression analysis are shown in Supplementary Information (including Figs. S1 and S2).

There were 357 participants who reported having had a PCR-confirmed infection by the 2022 health check-up. N-specific antibody results with respect to PCR-confirmed COVID-19 history are shown in Table 2. Among 669 seropositive participants, 325 (48.6%; 95% CI [42.7–50.3]) had no reported PCR-confirmed COVID-19 history. There were another 40 asymptomatic cases in the 344 cases that had a PCR-confirmed history of COVID-19. When adding these cases together, a total of 365 or 54.6% of 699 seropositive cases (95% CI [50.7–58.4]) were found to be asymptomatic. Although limited, we found 13 or 0.4% of 3119 seronegative participants (95% CI [0.2–0.7]) who reported a PCR-confirmed COVID-19 history but tested seronegative.

**Table 2:** N-specific antibody results (positive vs. negative) by confirmed previous COVID-19 infections (N = 3788).

	Overall	Total	N-specific antibody-positive, n (%)				
			No. of vaccine doses received				
			0 dose	1 dose	2 doses	3 doses	4 doses
All	3788 (100.0)	669 (17.7)	18 (2.7)	4 (0.6)	77 (11.5)	570 (85.2)	0 (0.0)
Without a confirmed COVID-19 history (a)	3431 (90.6)	325 (48.6)	4 (22.2)	1 (25.0)	32 (41.6)	288 (50.5)	0 (0.0)
With a confirmed COVID-19 history	357 (9.4)	344 (51.4)	14 (77.8)	3 (75.0)	45 (58.4)	282 (495)	0 (0.0)
Symptomatic	314	304	14	3	44	243	0
Asymptomatic (b)	43	40	0	0	1	39	0
(a) + (b) Asymptomatic	3474 (91.7)	365 (54.6)	4 (22.2)	1 (25.0)	33 (42.9)	327 (57.4)	0 (0.0)

	Overall	Total	N-specific antibody-negative, n (%)				
			No. of vaccine doses received				
			0 dose	1 dose	2 doses	3 doses	4 doses
All	3788 (100.0)	3119 (82.3)	62 (2∙0)	10 (0.3)	223 (7.2)	2814 (90.2)	10 (0.3)
Without a confirmed COVID-19 history (a)	3431 (90.6)	3106 (99.6)	61 (98.4)	10 (100.0)	223 (100.0)	2802 (9.6)	10 (100.0)
With a confirmed COVID-19 history	357 (9.4)	13 (0.4)	1 (1.6)	0 (0.0)	0 (0.0)	12 (0.4)	0 (0.0)
Symptomatic	314	10	1	0	0	9	0
Asymptomatic (b)	43	3	0	0	0	3	0
(a) + (b) Asymptomatic	3474 (91.7)	3109 (99.7)	61 (98.4)	10 (100.0)	223 (100.0)	2805 (99.7)	10 (100.0)

While no specific variant information of the infected cases was available in this study, of all 357 PCR-confirmed COVID-19 cases by year 2022 (Fig. 1b), 282 cases (79.0%; 95% CI [74.4–83.1] were found after December 26, 2021, when B.1.1.529 (Omicron variant) was initially detected in JUH. Notably, among the 282 infected HCW participants, 269 (95.3%) had either received two or three doses of COVID-19 vaccine.

## Discussion

This cohort study conducted in a Japanese frontline medical center, where most HCWs received three vaccine doses, showed that the seroprevalence of infection at the annual health check-ups of HCWs rose from 0.3 to 17.7% during 2020–2022. Since PCR-confirmed COVID-19 cases, with data on confirmation dates, increased rapidly immediately after the Omicron variant emerged in JUH as in the Tokyo metropolitan community, these infections were considered predominantly the Omicron variant. Yet, the surge of seroprevalence in the HCWs of JUH in 2022 was still similar or lower than earlier reports in other countries^17–19^, even though serological testing was conducted about six months after the first detection of the Omicron variant in JUH. This may be attributed to strict infection control measures at JUH, as well as the wearing of masks and maintenance of social distancing outside workplaces in Japan.

Of the HCWs with seropositive results, 48.6% had no reported PCR-confirmed infection history. Due to our strict infection control protocols and mandated PCR tests for HCWs with symptoms indicative of COVID-19 or close contacts of confirmed cases, these cases can be considered as SARS-CoV-2 infection without awareness. If adding the number of asymptomatic PCR-confirmed infection cases, 54.6% of the N-seropositive participants had no symptoms. Our findings are consistent with the previous research in a medical center including both HCWs and patients with booster doses during the period of Omicron variant dominance^10^. Other previous studies found that individuals who received booster doses had symptoms with SARS-CoV-2 infections less frequently and were more likely to be asymptomatic^20,21^. Other studies indicated that asymptomatic or mildly symptomatic SARS-CoV-2 infections would cause community transmission^6,22,23^. Not only the high transmissibility of the Omicron variant, but also unawareness of infections, might have caused person-to-person transmission and contributed to the surge of seroprevalence in 2022 at JUH.

This study has some limitations. First, it was conducted in a single medical center with a high vaccination rate and younger, more female HCWs; therefore, interpretation of the present study results should be done cautiously when applied them to the general population of Tokyo. Second, the symptoms of SARS-CoV-2 infection were self-reported by individuals and not verified by objective measures. Third, data on specific variants for infected cases were not available in this study.

The present study showed that, from 2020 to 2022, SARS-CoV-2 infection increased rapidly among Japanese HCWs, especially after the onset of the Omicron variant. Infections without awareness might be the key causal factor for rapid person-to-person transmission in this highly vaccinated medical center. Real-world evidence showed that through vaccination and natural infection, HCWs are likely to have lighter or no symptoms when infected with SARS-CoV-2 in the future^24–26^. A similar trend is expected to be seen in Japan. On the other hand, COVID-19 remains a threat to the public in Japan due to its strong impact on high-risk populations, including the large number of elderly who frequently visit hospitals and/or need inpatient medical care. Thus, preventing infection without awareness among HCWs and consequently preventing possible nosocomial infections is critical in medical settings.

Although it might be difficult to completely control spread in medical facilities, continued efforts, including routine temperature testing, maintenance of good hygiene habits and universal masking, continue to be required in medical settings even after some countermeasures (including wearing face masks) are lifted publicly in Japan. In addition, flexible policy changes, such as increasing the frequency of PCR testing for frontline HCWs in communities with high levels of prevalence, are important for detecting asymptomatic infection and halting transmission among HCWs^27,28^.

## Methods

### Description of infection control measures for HCWs

At our hospital, since the start of the COVID-19 pandemic, strict infection control measures have been certified by the Joint Commission International (JCI)^29^. Masking, in addition to face shield or eye protection, is universally required while in the hospital. In addition, using N-95 respirators is mandated when caring for patients with suspected or confirmed COVID-19. Daily temperature checks take place onsite at the workplace, with suspicious symptoms of COVID-19 requiring further examination. PCR tests are required for HCWs with symptoms indicative of COVID-19 or close contacts of confirmed cases. Dining with more than three non-family members outside workhours is discouraged in accordance with hospital policy.

### Study design and participants

In this cohort study, HCWs in Juntendo University Hospital (JUH) who participated in annual health checkups in 2022 at the university were recruited and gave consent for serological analysis. HCWs included medical doctors, nurses, laboratory personnel, paramedics, administrative staff, researchers, and others. As a frontline hospital, there are medical staff who work in COVID-19 wards and receive an extra allowance for the risk of exposure to SARS-CoV-2 infection in their work; they were defined as “COVID-19-dedicated staff” in this study.

The serum samples at the health checkup were collected, and the vaccination records and COVID-19 infection history were extracted from the hospital’s electronic records system. N-specific and S-specific antibodies are expected to be generated in individuals infected with SARS-CoV-2^30^. The currently available vaccines in Japan, including the mRNA vaccines (BNT162b2 and mRNA-1273) used for JUH’s vaccination campaigns, do not generate N-specific antibodies, but S-specific antibodies^31^. Thus, participants with seropositive results with N-specific antibodies can be considered previously infected with SARS-CoV-2. Those with insufficient amounts of serum samples to measure N-specific antibody titers were excluded. Titers of N-specific antibodies were measured using serological assays (Roche Diagnostics, Basel, Switzerland).

In this study, we measured blood samples of 3,788 HCWs at their 2022 health check-ups. We also compared the seroprevalence of 2022 to that of the past two years, which had been previously reported from our studies as 0.3% (14 out of 4,147 participants) in year 2020 and 1.6% (35 out of 2,202 participants) in 2021, respectively^12,13^. Each year’s number of study participants are different due to staff changes, participants’ consent for using their blood samples and targeted study population (for year 2021, only HCWs who had received 2 doses of COVID-19 vaccine by the time of 2021 health-check-ups were included). The time periods of annual health check-ups and vaccination campaigns from 2020 to 2022 at JUH are presented in Fig. 1b.

This study complied with all the relevant national regulations and institutional policies and was performed in accordance with the tenets of the Declaration of Helsinki. Informed consent was obtained from all study participants. This study was approved by the Institutional Review Board (IRB) of Juntendo University Hospital (IRB #M20-0089-M01).

### Measurement of SARS-CoV-2 antibodies

The Elecsys Anti-SARS-CoV-2 (Roche Diagnostics) immunoassay was used with the Cobas e801 analyzer approved by the U.S. Food and Drug Administration to measure SARS-CoV-2 N-specific antibody concentrations in accordance with the manufacturer’s instructions. The Elecsys Anti-SARS-CoV-2 immunoassay is used to detect N-specific total antibodies and has sensitivity of 100% and specificity of 99.8% (≥ 14 days after PCR diagnosis)^32^. The results are reported as numeric values in the form of a cut-off index (COI; signal sample/cutoff) with qualitative results. A COI ≥ 1.0 is interpreted as a positive test result.

### Statistical analyses

The seroprevalence in the HCWs of JUH was analyzed by age category, sex, vaccination status, professional category, and whether they were COVID-19-dedicated staff in 2022. Furthermore, in N-seropositive participants in 2022, the percentage of unaware infections was calculated by dividing the number of cases without PCR-confirmed infections by the total number of N-seropositive cases.

The seroprevalence is presented as crude percentages with 95% CIs. To evaluate the differences in seroprevalence by basic characteristics, Chi-squared tests or Fisher’s exact tests were performed to compare basic characteristics between groups, and Fisher’s exact tests were performed among profession categories and vaccination statuses followed by Bonferroni’s multiple comparison test.

Univariable logistic regression analysis was performed to compute ORs of seroprevalence with respect to basic characteristics. Then, multivariable logistic regression analysis was performed with an adjusted model to compute ORs of seroprevalence in COVID-19-dedicated staff. As for age categories, those aged 60–69 years and 70 years or older were combined when computing ORs because of their small sample sizes. A two-tailed *p* < 0.05 was considered significant. Statistical analysis was performed using R statistical software version 4.2.0. (Supplementary Information).

## Supplementary Information

Supplementary Information.

## Data Availability

All data used in the study are shown in the figures and tables. There are no more data to disclose.
