# Supplementary material for: Increased SARS-CoV-2 seroprevalence and spread of infection without awareness among healthcare workers through 2020–2022 in a Japanese medical center
Source: Sci Rep. 2023 Mar 27;13:4941. doi: 10.1038/s41598-023-32193-4 (PMC10040914; doi:10.1038/s41598-023-32193-4)
Supplement: Supplementary file 1 — Supplementary Information. [file 41598_2023_32193_MOESM1_ESM.pdf]

# Supplementary Information

**Article:** Increased SARS-CoV-2 seroprevalence and spread of infection without awareness among healthcare workers through 2020-2022 in a Japanese medical center

**Authors:** Rie Kanamori, Yan Yan, Kanami Ito, Hiroshi Fukuda, Satoshi Hori, Takamasa Yamamoto, Gene Igawa, Kaori Saito, Yuki Horiuchi, Shuko Nojiri, Yuji Nishizaki, Yoko Tabe, Kazuhisa Takahashi, Toshio Naito

**Contents:**

1. Table S1. Number of participants by profession and age categories.
2. Table S2. Number of participants of COVID-19 dedicated staff and profession categories.
3. Supplementary information regarding analysis of seroprevalence with respect to the HCW participants' characteristics
4. Figure S1. ORs of SARS-CoV-2 infection respect to participants' basic characteristics
5. Figure S2. ORs of seroprevalence on multivariable logistic regression analysis.

1. **Table S1. Number of participants by profession and age categories.**

| Age (years) | Overall | Profession category | | | | | | |
|---|---|---|---|---|---|---|---|---|
| | No. of participants | Medical Doctors | Nurses | Laboratory personnel | Paramedics | Administrative staff | Researchers | Others |
| **All** | 3788 (100·0) | 1497 (100·0) | 1080 (100·0) | 182 (100·0) | 320 (10·0) | 542 (10·0) | 157 (100·0) | 10 (100·0) |
| **20-29** | 1092 (28·8) | 255 (17·0) | 570 (52·8) | 63 (34·6) | 73 (22·8) | 93 (17·2) | 31 (1·7) | 7 (70·0) |
| **30-39** | 1171 (31·0) | 499 (33·3) | 297 (27·5) | 49 (26·9) | 108 (33·8) | 170 (31·4) | 46 (29·3) | 2 (20·0) |
| **40-49** | 829 (21·9) | 369 (26·5) | 145 (13·4) | 27 (14·8) | 71 (22·2) | 150 (27·7) | 400 (25·5) | 0 (0·0) |
| **50-59** | 436 (11·5) | 201 (13·4) | 54 (5·0) | 26 (14·3) | 41(12·8) | 91 (16·8) | 23 (14·6) | 0 (0·0) |
| **60-69** | 212 (5·6) | 113 (7·5) | 12 (1·1) | 16 (8·8) | 25 (7·8) | 31 (5·7) | 14 (8·9) | 0 (0·0) |
| **70-** | 48 (1·3) | 33 (2·2) | 2 (0·2) | 1 (0·5) | 2 (0·6) | 7 (1·3) | 3 (1·9) | 1 (10·0) |

2. **Table S2. Number of participants of COVID-19 dedicated staff and profession categories.**

| | Overall | COVID-19 dedicated staff |
|---|---|---|
| | No. of participants | No. of participants |
| **All** | 3788 (100.0) | 855 (22.6) |
| **Medical Doctors** | 1497 (100.0) | 423 (28.3) |
| **Nurses** | 1080 (100.0) | 283 (26.2) |
| **Laboratory personnel** | 182 (100.0) | 117 (64.2) |
| **Paramedics** | 320 (100.0) | 32 (10.0) |
| **Administrative staff** | 542 (100.0) | 0 (0.0) |
| **Researchers** | 157 (100.0) | 0 (0.0) |
| **Other** | 10 (100.0) | 0 (0.0) |

## 3. Supplementary information regarding analysis of seroprevalence with respect to the HCW participants' characteristics

### Methods

Univariable logistic regression analysis was performed to compute odds ratios (ORs) of seroprevalence with respect to basic characteristics. Then, multivariable logistic regression analysis was performed with an adjusted model to compute ORs of seroprevalence in COVID-19-dedicated staff. As for age categories, those aged 60-69 years and 70 years or older were combined when computing ORs because of their small sample sizes. A two-tailed p<0.05 was considered significant. Statistical analysis was performed using R statistical software version 4.2.0. *(also described in the main text, under Statistical Analysis section)*

### Results

Compared with the age group of 20-29 years, significantly lower ORs of seroprevalence were found across different age categories, except for the age group of 30-39 years: 0.85 (95%CI, 0.69–1.04; p=0.116) for 30–39 years; 0.78 (95%CI, 0.62–0.98; p=0.031) for 40–49 years; 0.46 (95%CI, 0.33 - 0.64; p<0.001) for 50-59 years; and 0.19 (95%CI, 0.11–0.34; p<0.001) for 60 years or older (**Supplementary Figure S1a**). ORs were not significantly different between male and female participants (**Supplementary Figure S1b**). As for different profession groups, a significantly higher OR was found in nurses (OR, 1.23; 95%CI, 1.02–1.50; p=0.034) compared with medical doctors, but ORs in laboratory personnel, paramedics, and administrative staff were significantly lower, at 0.48 (95%CI, 0.29 – 0.79; p=0.004), 0.50 (95%CI, 0.34–0.74; p<0.001), and 0.75 (95%CI, 0.57–0.98; p=0.034), respectively (**Supplementary Figure S1c**). There was no significant difference in ORs across different vaccination status groups compared to the unvaccinated group. ORs of 2 doses and 3 doses were 1.19 (95%CI, 0.66–2.14; p=0.561) and 0.70 (95%CI, 0.41–1.19; p=0.185), respectively (**Supplementary Figure S1d**).

Since younger age had a higher OR of seroprevalence, as shown in Supplementary Figure S1a, multivariable logistic regression analysis was performed including age category as a covariable. The OR of COVID-19-dedicated staff was not significantly different from that of non-COVID-19-dedicated staff in the adjusted model (p=0.222) (**Supplementary Figure S2**).

### Discussion

Regarding other causal factors, the infection rate of male and female participants was similar, consistent with our previous studies.[1,2] Nevertheless, the age group of 20-29 and nurses had higher odds ratios (ORs) than other categories. These factors are considered to be associated with each other, as shown in Supplementary Table S1. The present findings are consistent with previous research involving HCWs that showed nurses having higher seroprevalence or adjusted ORs compared to physicians.[3,4] On the other hand, the result that COVID-19-dedicated staff, consisting of several professions, was not associated with infection after adjusting for the effect of age, is consistent with our previous study.[1] These findings show that even though close and repeated contacts with infected patients may be a risk factor, the risk could be mitigated by compliance with the strict

infection control measures at medical facilities.

## References


1. Fukuda, H. et al. SARS-CoV-2 seroprevalence in healthcare workers at a frontline hospital in Tokyo. Sci Rep. **11**(1), 8380 (2021).
2. Igawa, G.et al. Antibody response and seroprevalence in healthcare workers after the BNT162b2 vaccination in a University Hospital at Tokyo. Sci Rep, **12**(1), 8707 (2022).
3. Poletti, P. et al. Seroprevalence of and risk factors associated with SARS-CoV-2 infection in health care workers during the early COVID-19 pandemic in Italy. JAMA Netw Open. **4** (7), e2115699 (2021). doi: 10.1001/jamanetworkopen.2021.15699.
4. Plebani, M. et al. SARS-CoV-2 serosurvey in health care workers of the Veneto Region. Clin Chem Lab Med. **58**(12), 2107-2111 (2020).

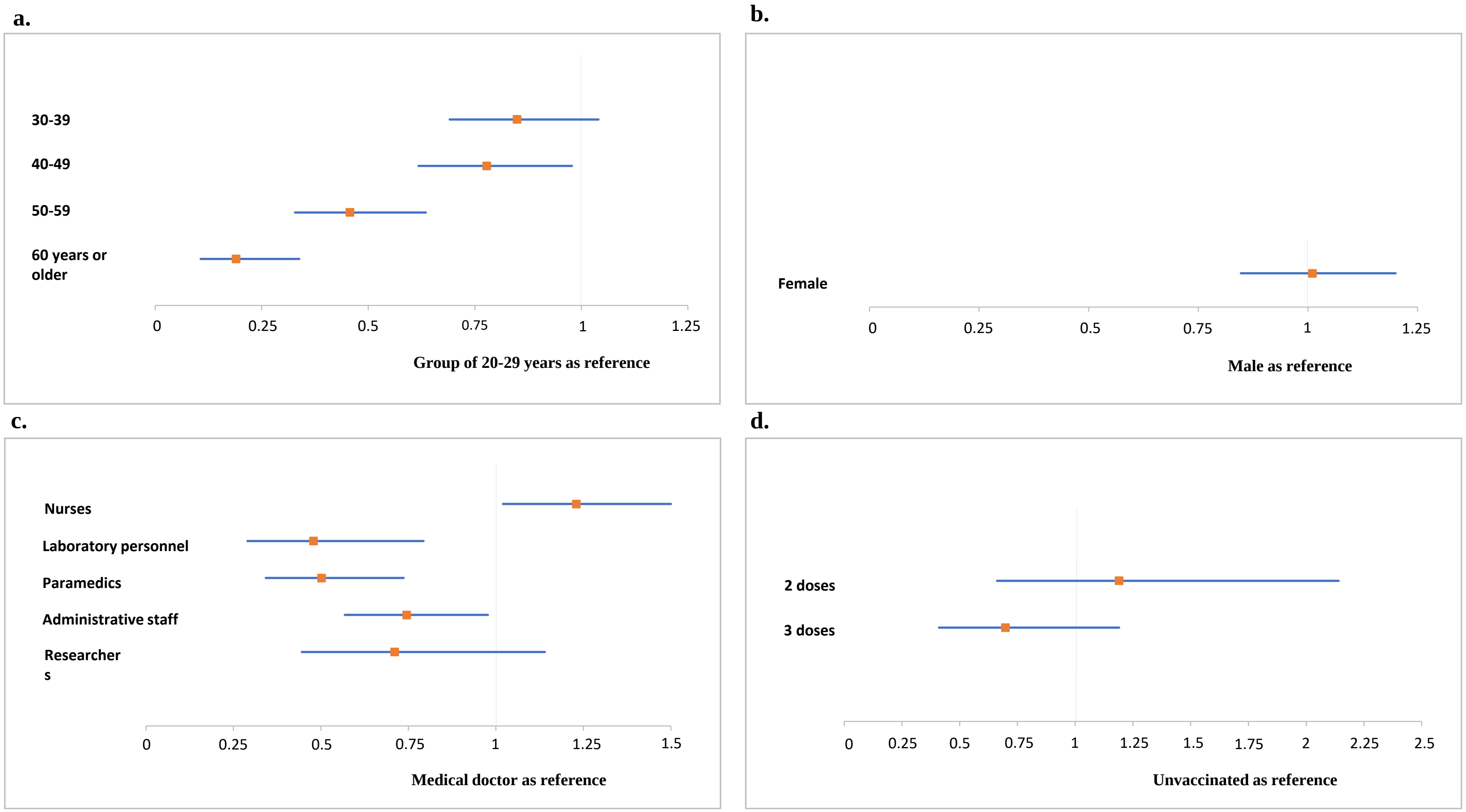


## 4. Figure S1. ORs of SARS-CoV-2 infection respect to participants' basic characteristics

a. ORs of seroprevalence by age category, with age 20-29 years as the reference.
b. ORs of seroprevalence by sex, with male as the reference.
c. ORs of seroprevalence by profession category, with medical doctor as the reference.
d. ORs of seroprevalence by vaccination status, with unvaccinated as the reference. The number of HCW participants who received 1 dose or 4 doses were 14 and 10, respectively, at the time of the health check-up of 2022; the two groups are not shown in Supplementary Figure 1d due to the small numbers.

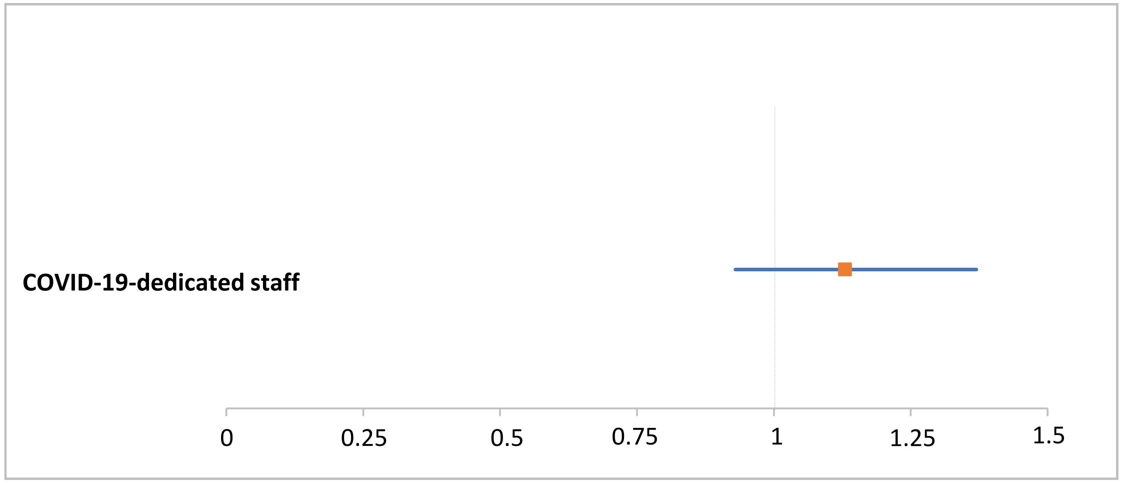


| Multivariable-adjusted ORs (95%CI) |
|---|
| 1.13 (0.93-1.37) |

**5. Figure S2. ORs of seroprevalence on multivariable logistic regression analysis.**

The multivariable model includes adjustment for staff group and age category.
